# Cobb angle outcomes in adolescent idiopathic scoliosis curves above the seventh/eighth thoracic vertebrae: A comparison of Milwaukee and Chêneau bracing techniques

**DOI:** 10.1016/j.xnsj.2026.100924

**Published:** 2026-06-26

**Authors:** Taher Babaee, Zahra Hedayati, Fateme Eskandarighargheh, Pir-Hossein Kolivand, Vahideh Moradi

**Affiliations:** a Department of Orthotics and Prosthetics, School of Rehabilitation Sciences, Iran University of Medical Sciences, Mirdamad Blvd, Shahnazari Street, Nezam Avenue, Tehran, Iran; b Department of Physical Therapy, University of Nevada, 4505 S Maryland Pkwy, Las Vegas, NV, 89154, USA; c Department of Health Economics, School of Medicine, Shahed University, Tehran, Iran; d Research Center for Health Management in Mass Gathering, Red Crescent Society of the Islamic Republic of Iran, Italia Avenue, Palestine St., Keshavarz Blvd., Valiasr square, Tehran, 1599845313, Iran

**Keywords:** Cobb angle, Adolescent idiopathic scoliosis, Brace, Orthotic treatment, Curve correction, Chêneau brace

## Abstract

**Background:**

Although the Chêneau brace is effective for 3-dimensional scoliosis correction, current expert recommendations generally limit its use, along with other thoracolumbosacral orthoses, to thoracic curves with an apex below T7/T8. The purpose of this study was to compare the effects of Milwaukee and Chêneau braces on Cobb angle and other radiographic parameters in adolescents with idiopathic scoliosis (AIS) presenting with thoracic curve apices above T7/T8.

**Methods:**

In this prospective comparative study, participants were treated with either a Milwaukee brace or a Chêneau brace for thoracic scoliosis with curve apices above the T7/T8 level. Both interventions incorporated brace-specific exercise programs designed according to the biomechanical principles of each orthosis. Clinical and radiographic assessments included sex, age, primary Cobb angle at brace initiation and best in-brace correction, T1–T12 kyphosis angle, trunk shift, apical vertebral translation, and coronal deformity angular ratio (C-DAR).

**Results:**

Fifty-two adolescents with AIS (12 males and 40 females) were enrolled. Both brace groups demonstrated significant improvements in scoliosis Cobb angle, thoracic kyphosis, trunk shift, and apical vertebral translation compared with pretreatment values (p < .05). However, no significant differences were observed between the Milwaukee and Chêneau groups regarding in-brace radiographic outcomes (p > .05).

**Conclusions:**

Both Milwaukee and Chêneau braces appear to be effective treatment options for adolescents with idiopathic scoliosis and thoracic curve apices above T7/T8, suggesting that Chêneau bracing may also be applicable in high thoracic curves traditionally managed with Milwaukee braces.

## Introduction

Adolescent idiopathic scoliosis is a 3-dimensional spinal deformity characterized by lateral deviation in the frontal plane, vertebral rotation in the transverse plane, and a loss of normal thoracic kyphosis in the sagittal plane [1]. Various treatment methods for scoliosis include physical therapy and exercise, bracing, and surgery. Of these, studies indicate that bracing plays an important role in the nonsurgical treatment of adolescents with idiopathic scoliosis measuring 15 to 60 degrees [2], with the goal of preventing curve progression during the growth spurt [3].

The selection of a brace for treating adolescent idiopathic scoliosis includes the use of thoracolumbosacral orthoses and the Milwaukee brace, which is determined by the location of the curve apex [4]. The Milwaukee brace is indicated for thoracic and double major curves with an apex at or above the T7/T8 vertebrae, whereas thoracolumbosacral orthoses are prescribed for single or double lumbar and thoracic curves with an apex below T7/T8 [1]. Although scoliosis affects the spine in all 3 planes of movement, most braces provide correction primarily in the frontal plane [5] and the greatest improvement in the Cobb angle does not always align with the greatest correction of vertebral rotation [6,7]. The Chêneau brace is one of the few designed to simultaneously correct deformities in all 3 planes [8]. Its design as a thoracolumbosacral orthosis for treating idiopathic scoliosis utilizes pressure zones and expansion areas to achieve comprehensive 3-dimensional correction [9,10]. However, despite the effectiveness of this brace in correcting scoliosis across all 3 planes, expert consensus and specialized guidelines are based on the assumption that this brace, along with other thoracolumbosacral orthoses, should only be used for thoracic curves with an apex below T7/T8 [[11], [12], [13]].

Although the Milwaukee brace has long been considered the preferred orthotic design for thoracic curves with an apex above T7/T8 [1], limited supporting data is available regarding its comparative effectiveness in relation to lower profile TLSOs, particularly the Chêneau brace, in adolescents with idiopathic scoliosis. Additionally, studies comparing the Milwaukee and Chêneau braces for high thoracic curves are lacking. Therefore, assessing whether the Chêneau brace can obtain corrective outcomes comparable to those of the Milwaukee brace in scoliosis curves with an apex above T7/T8 appears warranted. This belief originates from the argument that the trimlines of the TLSOs such as the Chêneau brace are designed in a way that it cannot apply corrective forces to curves above T7/T8 [14]. It is important to note that the efficacy of the Milwaukee brace for treating higher thoracic curves is not well-established and no studies have specifically investigated this issue. Also, numerous studies comparing the Milwaukee brace with TLSOs have reported that the use of TLSOs has a less negative impact on adolescents’ overall quality-of-life scores, particularly with respect to their psychosocial functioning and body image [4,15]. Therefore, considering the reported adverse effects associated with the Milwaukee brace and the lack of studies investigating the efficacy of this brace—particularly the Chêneau brace—for curves with an apex above T7/T8, conducting a study on this subject appears essential. Given the effectiveness of the Chêneau brace and its upper trim line extending to the level of the fifth thoracic vertebra, we hypothesize that this brace may also be effective for curves with an apex above the T7/T8 vertebrae. Therefore, the present study aims to compare the short-term effects of Milwaukee and Chêneau braces on adolescent idiopathic scoliosis curves above the T7/T8 vertebrae.

## Materials and methods

### Study design and participants

The current study is a prospective comparative study. The clinical and radiological data from adolescents with idiopathic scoliosis who were prescribed either the Milwaukee or Chêneau brace were collected between March 2024 and June 2025 in 3 spine clinics in Tehran, Iran. We conducted this study in compliance with the principles of the Declaration of Helsinki. The study’s protocol was reviewed and approved by the Institutional Review Board of the Iranian Red Crescent Society (Approval No. IR.RCS.REC.1403.025). Written informed consents were obtained from the families of the participants. The inclusion criteria of the study were set according to the guidelines of the Scoliosis Research Society for brace treatment [2,16]: adolescent idiopathic scoliosis cases with a referral age between 10 and 15 years; pre-brace Risser sign grade of 0 to 2; Cobb angle of 20° to 50° at initiation of brace treatment; no previous treatment; if female, either premenarchal or less than 1 year postmenarchal; and existing the whole radiographic details from initiation of brace treatment up to a minimum 6 months after start of brace treatment. All adolescent idiopathic scoliosis cases with compliance rate of <75%, lacking clinical or radiographic data through brace treatment and follow-up and those who stopped brace treatment were excluded [17]. All braces were manufactured by a certified orthotist based on the prescription of a spine surgeon.

### Brace design and treatment protocol

**Fig. 1:**
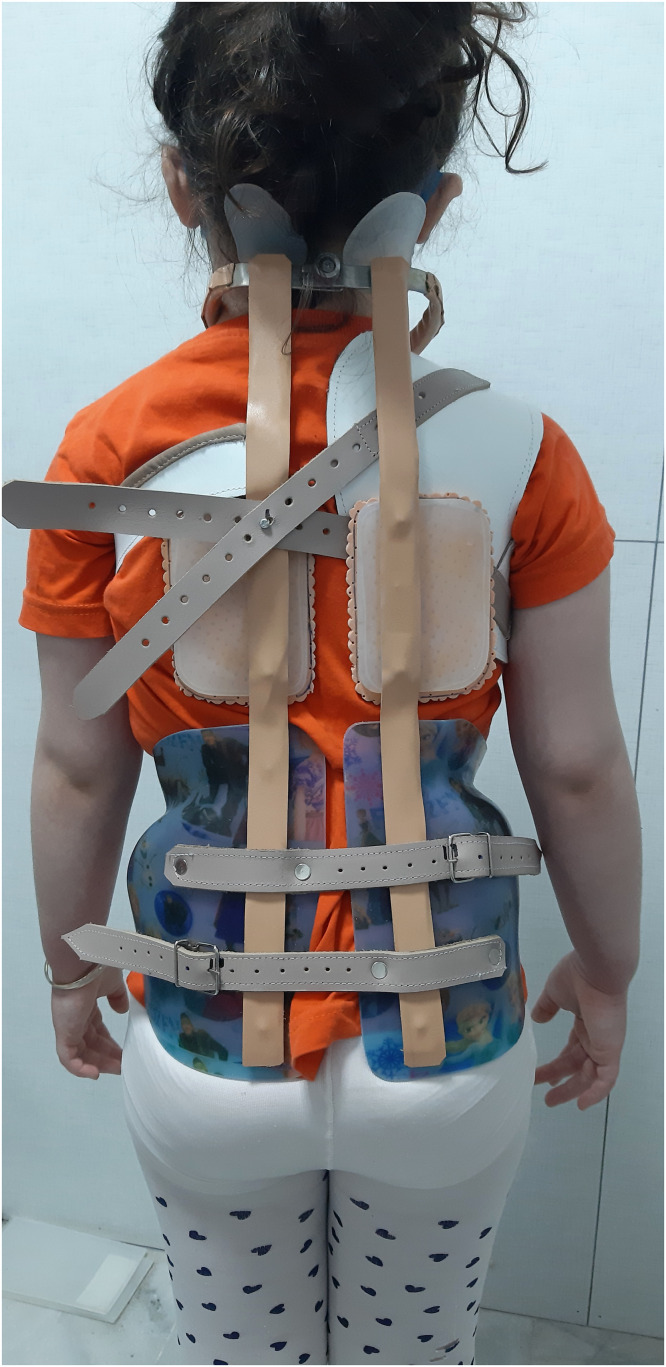
The Milwaukee brace.

**Fig. 2:**
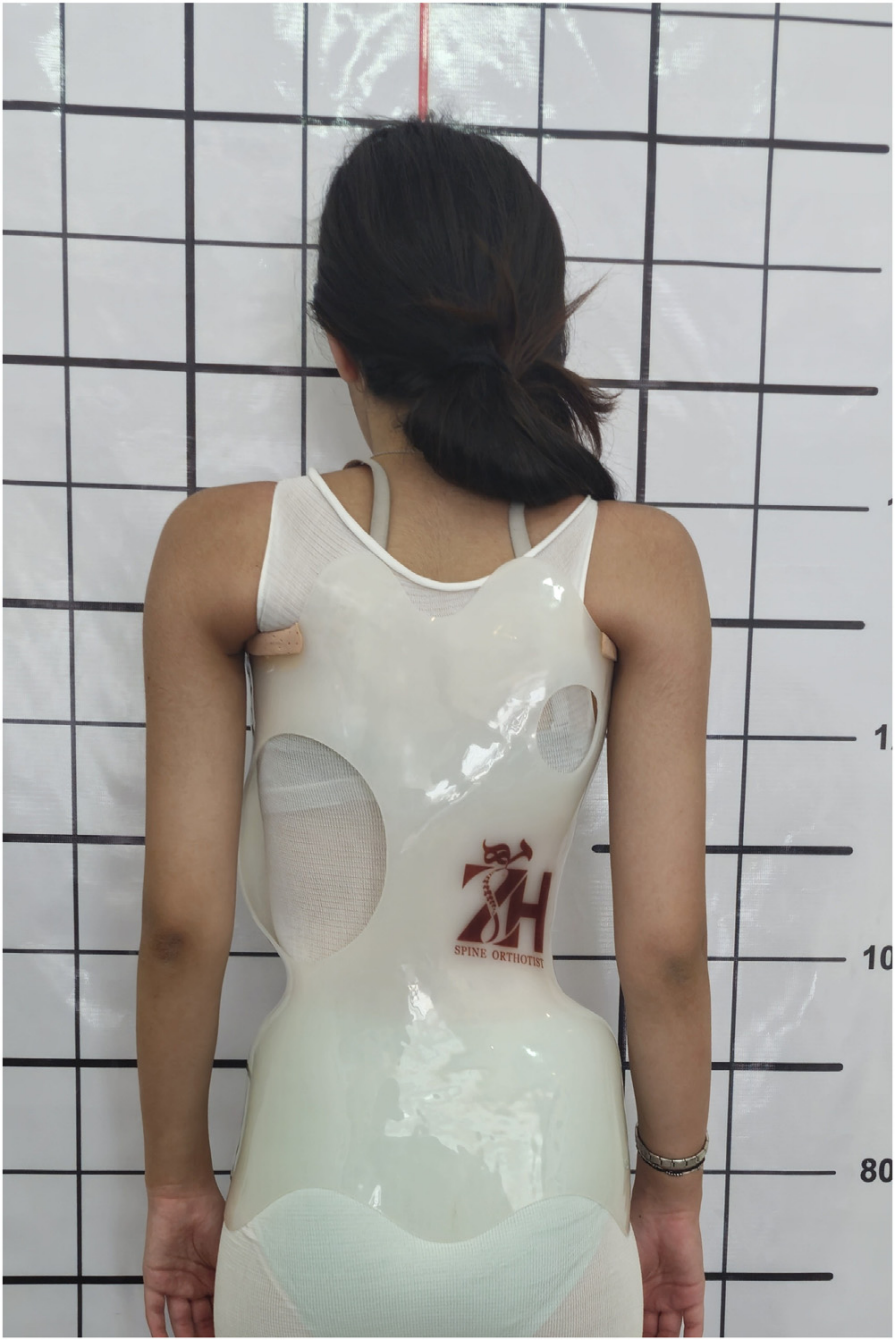
The Chêneau brace.

Participants were treated with either a Milwaukee brace (Fig. 1) or a Chêneau brace (Fig. 2), both prescribed for thoracic scoliosis with curve apices located above the T7/T8 vertebrae. Each intervention combined a specific brace design with a structured exercise protocol tailored to its biomechanical correction principles. The Milwaukee brace used in this study consisted of a pelvic section connected to a neck ring by 1 anterior and 2 posterior uprights. Correction of the scoliosis was achieved through transverse forces applied by a thoracic pad or shoulder ring fabricated from low-density polyethylene and lined with foam to improve comfort and force distribution. This design aimed to control upper thoracic curves by maintaining axial elongation and limiting curve progression. Patients in the Milwaukee brace group were instructed to wear the brace for approximately 23 hours per day. In addition, they followed a standardized Blount and Moe exercise program [18] for a total of 2 hours daily, divided equally between in-brace and out-of-brace sessions. The exercise program was intended to preserve trunk muscle strength, support postural alignment, and reinforce the corrective effect of the brace. Exercises included pelvic tilting, active trunk shifting away from the thoracic pressure area, and coordinated deep breathing with thoracic expansion. Patients were also trained to perform backward reaching movements toward the posterior uprights to encourage spinal extension. Pelvic tilt exercises were practiced in various positions, including supine, transitional sit-up movements, push-up positions, and standing. All patients received written and illustrated instructions to promote consistent performance of the exercises.

The Chêneau brace was individually fabricated using 5-mm thermoplastic material and designed to provide 3-dimensional correction of scoliosis. Its biomechanical concept relies on the application of asymmetric pressure zones combined with expansion spaces, allowing active realignment of the spine in the coronal, sagittal, and transverse planes. This design not only applies corrective forces but also facilitates patient participation in postural correction. Patients treated with the Chêneau brace participated in a supervised Schroth-based exercise program delivered by experienced physiotherapists. The program included spinal stabilization, postural training, rotational breathing, and active 3-dimensional autocorrection [19]. During training sessions, patients were guided to actively achieve and maintain a corrected spinal position. Breathing exercises were integrated into postural correction, with emphasis on directed inhalation toward the concave side of the curve and controlled exhalation from the convex side, aiming to reduce vertebral rotation and thoracic asymmetry. Daily practice of postural correction exercises was encouraged to enhance body awareness and promote long-term neuromuscular adaptation, potentially supporting sustained brace effectiveness.

### Data extraction

The clinical and radiological parameters of the adolescents diagnosed with idiopathic scoliosis who were included in this study were systematically documented as follows: (1) sex, (2) age, (3) primary scoliosis curve Cobb angle measured at the initiation of brace treatment and at the point of optimal in-brace correction, (4) T5–T12 kyphosis angle recorded at the initiation of brace treatment and at the best in-brace correction, (5) type of prescribed brace, (6) trunk shift, (7) apical vertebral translation, and (8) coronal-deformity angular ratio (C-DAR) assessed at the onset of brace treatment. Best in-brace correction (IBC) was calculated as the percentage reduction in Cobb angle [20]:

$$IBC=\frac{Standing\;cobb\;angle\;\left(no\;brace\right)-Standing\;cobb\;angle\;\left(best\;in-brace\right)}{Standing\;cobb\;angle\;\left(no\;brace\right)}\times 100$$

The value of initial C-DAR is an important predictor for long-term outcomes of bracing in adolescent idiopathic scoliosis. In this study, to control this confounding parameter, we matched the participants of 2 groups regarding the initial C-DAR. The C-DAR was calculated by dividing the Cobb angle of the primary curve by the number of vertebrae that displaced laterally in coronal plane [21].

Trunk shift was defined as the horizontal distance between a vertical line dropped from the seventh cervical vertebra (C7 plumb line) and the central sacral vertical line (CSVL), measured in centimeters [22]. Apical vertebral translation was measured in centimeters as the perpendicular distance from the center of the apical vertebra to the CSVL [22].

### Statistical analyses

Data analysis was conducted using SPSS (version 20.0; IBM Corp.). The normality of data distribution was assessed using the Kolmogorov–Smirnov test. The kyphosis angle satisfied a normal distribution, whereas the remaining parameters did not meet the assumption of normality. Accordingly, baseline characteristics at the initiation of brace treatment were compared between the Milwaukee and Chêneau brace groups using the independent-samples t-test for the kyphosis angle and the Mann–Whitney U test for the remaining parameters. For comparing pre-brace and in-brace measurements, the paired-samples t-test was used for the kyphosis angle and the Wilcoxon signed-rank test for the other parameters. The association between Risser grade and the magnitude of in-brace correction was evaluated using Spearman’s rank correlation coefficient. A p-value <.05 was considered statistically significant.

## Results

A total of 52 adolescents with AIS (12 males and 40 females) were included in the study. Of these, 25 patients were treated with a Milwaukee brace (mean age, 11.92 ± 1.07 years) and 27 patients with a Chêneau brace (mean age, 12.03 ± 1.15 years). At the initiation of brace treatment, a statistically significant difference was observed in Risser sign between the 2 groups (p = .02). However, there were no significant differences in age, scoliosis Cobb angle, kyphosis Cobb angle, C-DAR value, trunk shift, and apical vertebral translation between the 2 groups (p < .05) (Table 1).

**Table 1:** Baseline characteristics of the included participants between the Milwaukee brace and Chêneau brace.

Parameters	Milwaukee brace	Chêneau brace	Effect size	p-value
	Mean (SD)	Mean (SD)		
Age (y)	11.92 (1.07)	12.03 (1.15)	0.07	.63
Scoliosis Cobb angle (°)	29.60 (5.97)	33.00 (6.73)	0.23	.10
Kyphosis Cobb angle (°)	43.61 (13.05)	44.10 (11.87)	0.07	.80
Risser sign grade	1.32 (0.80)	0.81 (0.73)	0.32	.02
C-DAR	5.46 (1.72)	5.87 (1.90)	0.11	.41
Trunk shift (cm)	15.20 (5.85)	18.66 (5.26)	0.26	.05
Apical vertebral translation (cm)	33.72 (8.12)	34.85 (7.91)	0.06	.65

In the comparison of radiological parameters before and after brace treatment, the results related to the Milwaukee brace revealed statistically significant differences in scoliosis Cobb angle (p < .001), kyphosis Cobb angle (p = .01), trunk shift (p < .001), and apical vertebral translation (p < .001) compared to before using the brace (Table 2).

**Table 2:** Comparing baseline and in-brace radiographic values of patients under Milwaukee brace treatment.

Parameters	Baseline	In-brace	Effect size	p-value
	Mean (SD)	Mean (SD)		
Scoliosis Cobb angle (°)	29.60 (5.97)	15.95 (6.95)	0.86	<.001
Kyphosis Cobb angle (°)	44.80 (12.18)	37.85 (8.04)	0.58	.017
Trunk shift (cm)	15.20 (5.85)	5.07 (4.96)	0.85	<.001
Apical vertebral translation (cm)	33.72 (8.12)	16.08 (7.50)	0.87	<.001

In the Chêneau brace group, statistically significant differences were observed in scoliosis Cobb angle (p < .001), kyphosis Cobb angle (p < .001), trunk shift (p < .001), and apical vertebral translation (p < .001) when compared with before using the orthosis (Table 3).

**Table 3:** Comparing baseline and in-brace radiographic values of patients under Chêneau brace treatment.

Parameters	Baseline	In-brace	Effect size	p-value
	Mean (SD)	Mean (SD)		
Scoliosis Cobb angle (°)	33.00 (6.73)	19.25 (12.45)	0.85	<.001
Kyphosis Cobb angle (°)	44.10 (11.87)	37.29 (7.92)	1.18	.017
Trunk shift (cm)	18.66 (5.26)	7.18 (6.17)	0.87	<.001
Apical vertebral translation (cm)	34.85 (7.91)	16.85 (12.76)	0.85	<.001

In the comparison of in-brace radiological parameters between the Milwaukee and Chêneau brace groups, no statistically significant differences were observed in any of the evaluated radiological parameters (Table 4).

**Table 4:** Comparing the in-brace values of radiographic parameters between 2 groups.

Parameters	Milwaukee brace	Chêneau brace	Effect size	p-value
	Mean (SD)	Mean (SD)		
Scoliosis Cobb angle (°)	15.95 (6.95)	19.25 (12.45)	0.21	.12
Kyphosis Cobb angle (°)	37.85 (8.04)	37.29 (7.92)	0.07	.81
Trunk shift (cm)	5.07 (4.96)	7.18 (6.17)	0.19	.17
Apical vertebral translation (cm)	16.08 (7.50)	16.85 (12.76)	0.01	.90

To assess the potential influence of skeletal maturity on in-brace correction, the association between Risser grade and in-brace correction was examined separately in the Milwaukee and Chêneau groups. No significant relationship was observed between Risser grade and in-brace correction in either group (Milwaukee: *r* = 0.29, p = .13; Chêneau: *r* = .01, p = 0.93).

## Discussion

Bracing is commonly utilized to prevent curve progression of adolescent idiopathic scoliosis during periods of skeletal growth. Its effectiveness in controlling the progression of scoliosis curvature is well-documented and depends on parameters such as the location of the curve, severity of the deformity, patient age, flexibility of the curve, and the design of the selected brace [[23], [24], [25]]. Among commonly prescribed braces, the Milwaukee and Chêneau braces have established clinical efficacy but differ in their indications and underlying biomechanical principles. Despite this distinction, the Chêneau brace is frequently preferred by both patients and clinicians due to its lighter construction and better aesthetics, factors that may enhance adherence to treatment [26]. The Milwaukee brace is recognized as a standard treatment for thoracic and double curves, especially those above T8, due to its specific design that allows correction in both sagittal and coronal planes [1]. Nevertheless, evidence regarding the effectiveness of the Chêneau brace in higher thoracic curves remains limited. This study therefore provides a comparative evaluation of the Milwaukee and Chêneau braces in adolescents with idiopathic scoliosis curves above T7/T8, aiming to clarify their relative effectiveness within this specific patient population.

The findings of the present study demonstrated that both the Chêneau and Milwaukee braces lead to significant reductions in scoliosis Cobb angle, kyphosis Cobb angle, trunk shift, and apical vertebral translation after 6 months of brace use. The significant reduction in the Cobb angle indicates the high effectiveness of these braces in controlling and correcting lateral curvature of the spine. This result aligns with previous studies showing that these braces can effectively control the progression of scoliosis curvature [12,[27], [28], [29], [30]]. Additionally, maintaining the kyphosis Cobb angle within the normal range (20°–40°) [31] indicates the positive effects of both the Milwaukee and Chêneau braces on the sagittal plane. The reduction in trunk shift reflects improved balance and alignment of the spine in the frontal plane. Decreasing trunk shift can enhance the patient’s appearance and reduce asymmetric pressures on the spine. Furthermore, the reduction in apical vertebral translation indicates the positive impact of the Milwaukee and Chêneau braces in reducing apical vertebral displacement. This parameter is considered one of the important indicators in assessing the severity of scoliosis and the effectiveness of treatment [22].

Contrary to prior assumptions and studies, the Chêneau brace achieved meaningful correction even in curves with an apex above T7/T8. However, their findings emphasized that the greatest corrective effects were observed in lumbar and thoracolumbar curves [13,32]. Similarly, Pepke et al. found significant Cobb angle improvements in thoracic and lumbar regions, while no corrective effect was observed on upper thoracic vertebrae [11]. The findings related to the Milwaukee brace were consistent with previous studies, demonstrating its high effectiveness in correcting thoracic curves [28,33]. Notably, direct comparison revealed no significant difference in in-brace radiological parameters between the Milwaukee and Chêneau braces for curves with an apex above T7/T8. The absence of significant differences between the 2 groups may suggest that, in high thoracic curves, the magnitude of in-brace correction depends more on the overall ability of the brace to achieve effective trunk realignment than on the specific biomechanical strategy employed. Although the Milwaukee and Chêneau braces are based on distinct corrective concepts, both appear capable of generating sufficient corrective forces to produce comparable radiographic improvements. The Milwaukee brace primarily achieves correction through pelvic fixation, longitudinal distraction, and strategically positioned pads, whereas the Chêneau brace utilizes an asymmetric system of pressure and expansion zones to promote 3-dimensional correction and derotation [34]. Despite these structural differences, both braces may provide adequate control of trunk alignment and corrective loading of the thoracic spine, resulting in similar reductions in Cobb angle, trunk shift, and apical vertebral translation. This may explain why no significant differences in in-brace radiological outcomes were observed between the Milwaukee and Chêneau braces in the present study. To our knowledge, this finding has not been previously reported. The lack of a significant difference between 2 braces in curves above T7/T8 challenges the traditional perspectives regarding brace selection for this subgroup of patients. Although the Milwaukee brace remains a well-established and effective treatment option [35], our findings suggest that the Chêneau brace may also be considered an acceptable alternative for higher thoracic scoliosis curves. This finding is of noteworthy clinical relevance, as despite the well-established effectiveness of the Milwaukee brace in previous studies, brace-related adverse effects and discomfort remain significant factors contributing to reduced patient acceptance and compliance. The Milwaukee brace has been associated with reduced compliance and psychosocial disorders—including diminished self-esteem in adolescents—due to its bulky suprastructure and highly visible design [4,36]. Moreover, long-term follow-up studies have also reported complications such as temporomandibular joint disorders and psychosocial issues [37]. In this context, the Chêneau brace, with its lighter design and potentially higher treatment adherence, may serve as a suitable alternative for selected patients and provide clinicians with a more flexible therapeutic approach.

## Limitations

This study has several limitations that should be acknowledged. Although the sample size is sufficient for preliminary findings, it may not fully encompass the range of scoliosis curve variations and individual responses to treatment. In addition, even though in-brace curve correction is considered one of the most important predictors of treatment effectiveness in adolescent idiopathic scoliosis [25], the lack of long-term follow-up data limits our ability to draw conclusions regarding sustained effectiveness and potential brace-related complications. Future research should include larger, multicenter studies to evaluate the long-term outcomes of both braces regarding curve progression, quality of life, and psychosocial impacts. Furthermore, investigation of the biomechanical differences between the 2 braces may provide deeper insights into their mechanisms of action and help identify patient populations that are most likely to benefit from each brace type. Finally, although the groups differed significantly in baseline Risser grade, further analyses showed no significant relationship between Risser grade and in-brace correction. This finding is not unexpected, as in-brace correction reflects the immediate effect of the brace and may be less dependent on skeletal maturity than long-term treatment outcomes such as treatment success or failure.

## Conclusion

The findings of the present study suggest that both the Chêneau and Milwaukee braces may be considered effective treatment options for the management of adolescents with idiopathic scoliosis curves above T7/T8. Importantly, these results may contribute to a paradigm shift in clinical decision-making, as they indicate that the Chêneau brace—owing to its lighter design and potentially fewer adverse effects—may serve as a viable alternative for patients who experience limitations or reduced tolerance with the Milwaukee brace.

## Authors’ contributions

TB analyzed and interpreted the data. ZH gathered the data and wrote the first article draft. All authors read and approved the final manuscript.

## Funding

The authors disclosed that they received no financial support for the research, authorship, and/or publication of this article.

## Ethics approval

The study’s protocol was reviewed and approved by the Institutional Review Board of the Iranian Red Crescent Society (Approval No. IR.RCS.REC.1403.025).

## Patient informed consent statement

Written informed consents were obtained from the families of the participants.

## Availability of data and materials

The dataset used and/or analyzed during the current study are available from the corresponding author on reasonable request.

## Declaration of competing interests

The authors declare that they have no known competing financial interests or personal relationships that could have appeared to influence the work reported in this paper.
